# Critical Care Science - New title da *Revista Brasileira de Terapia Intensiva*

**Published:** 2023

This issue celebrates the first edition of Critical Care Science, previously known as *Revista Brasileira de Terapia Intensiva* (RBTI). For years, RBTI has been acknowledged in all Ibero-America, as a reference for clinicians and scientists in the field of critical care medicine. RBTI is the official journal of both the *Associação de Medicina Intensiva Brasileira* (AMIB) and the *Sociedade Portuguesa de Cuidados Intensivos* (SPCI). Since its indexation in different platforms, the journal has increased its reach in the international scientific community, and, over the last years, about 30% of the submitted manuscripts came from outside of Brazil.

During the last years, the editorial board and representatives from AMIB and SPCI discussed changing the original Portuguese title acknowledge this increasing diversity of authors. We believe that the change in the journals’ name reflects the trajectory of a journal that is well consolidated in Brazil and with broad international coverage. Our efforts are focused on making Critical Care Science a journal that reflects the current maturity and needs of the international scientific community. This change will improve its competitiveness and increase the submission of manuscripts, both in number and quality.

Despite the new name, Critical Care Science will maintain the same mission and uphold its longstanding scientific and ethical standards. The support of AMIB and SPCI in this context is essential. We wish to invite all the international critical care community to submit both basic and clinical research in this field to our journal. Besides its long history as a relevant vehicle in disseminating high-value research in the critical care field, Critical Care Science will continue the editorial policy of RBTI and will remain fully open access with no article processing charges.

This change has been an arduous task, but after years of editorial experience, and the support of AMIB and SPCI we are convinced that these changes will improve its visibility globally serving better our authors and readers, the supporting societies, and all the critical care scientific community.

**Editorial Board**

Critical Care Science

